# Burnout or anxiety?! A medical trial regarding resident doctors in a first line University Hospital during COVID 19 pandemic

**DOI:** 10.1192/j.eurpsy.2022.507

**Published:** 2022-09-01

**Authors:** C. Sapaniuc, G. Lacatusu, I. Mihai, M. Rascanu, D. Bran, D. Manciuc

**Affiliations:** 1 Hospital of Infectious Diseases “Sf. Parascheva”, Infectious Diseases, Iasi, Romania; 2 Universiity of Medicine and Pharmacy “Gr. T. Popa”, Infectious Diseases, iasi, Romania; 3 Hospital of Infectious Diseases “Sf. Parascheva”, Psychology, Iasi, Romania

**Keywords:** burnout, anxiety, pandemic, covid 19, resident doctor, hospital

## Abstract

**Introduction:**

Coronavirus Disease 2019 (COVID-19) caused by severe acute respiratory syndrome coronavirus 2 (SARS-CoV-2) has been reported as a worldwide emergency. Due to the extensiveness of spread and death, it has been declared as a pandemic.

**Objectives:**

To highlight how COVID-19 pandemic psycho-emotional affects the medical staff of a frontline University Hospital in the “battle” with new coronavirus.

**Methods:**

We employed a cross-sectional survey of 71 resident doctors from a frontline Hospital after a one-year pandemic and analyzed the prevalence and associated factors with work-related psychological distress among our study group.

**Results:**

Out of the hospital resident doctors, 71 participated and completed the questionnaire, offering an overall response rate of 100%. The majority of participants were women (86% - 61). The average age was 29 years. Most respondents were unmarried. A total of 67% of participants were non-smokers, 5% stated that they occasionally consumed alcohol, none of them used drugs. As a result of the qualitative and quantitative analysis of the data, aspects related to anxiety (21.12% - 15), exhaustion (15.49% - 11), and depression (11.26% - 8) are highlighted. In our study, no people were identified who would reach extreme exhaustion in the work process, due to good resilience and due to a well thought out program of work and rest during the pandemic.

**Conclusions:**

The psychological pressure at work, as well as the one felt after limiting and restricting mobility for shorter or longer periods, had an impact on the psycho-emotional state of health care workers, requiring further psychological reassessments and psychological support.

**Disclosure:**

No significant relationships.

